# The Borg family of Cdc42 effector proteins Cdc42EP1–5

**DOI:** 10.1042/BST20160219

**Published:** 2016-12-02

**Authors:** Aaron J. Farrugia, Fernando Calvo

**Affiliations:** Tumour Microenvironment Team, Division of Cancer Biology, Institute of Cancer Research, 237 Fulham Road, London SW2 6JB, U.K.

**Keywords:** Borg, Cdc42, cytoskeleton, Rho GTPases, septin

## Abstract

Despite being discovered more than 15 years ago, the Borg (binder of Rho GTPases) family of Cdc42 effector proteins (Cdc42EP1–5) remains largely uncharacterised and relatively little is known about their structure, regulation and role in development and disease. Recent studies are starting to unravel some of the key functional and mechanistic aspects of the Borg proteins, including their role in cytoskeletal remodelling and signalling. In addition, the participation of Borg proteins in important cellular processes such as cell shape, directed migration and differentiation is slowly emerging, directly linking Borgs with important physiological and pathological processes such as angiogenesis, neurotransmission and cancer-associated desmoplasia. Here, we review some of these findings and discuss future prospects.

## Introduction

The Rho GTPase family member Cdc42 regulates a diverse range of cellular functions including cytokinesis, cytoskeletal remodelling and cell polarity [1,2]. Like other Rho family members, Cdc42 cycles between two tightly regulated conformational states, a GTP-bound active state and a GDP-bound inactive state [3]. Activated Cdc42 exerts its functions by interacting with downstream effectors containing a Cdc42/Rac interactive binding (CRIB) motif [3,4]. Several Cdc42 effector proteins, including kinases and scaffolds, have been well characterised [5]; however, the functions of others remain relatively unknown. Still, elusive is the largely understudied Borg family of Cdc42 effector proteins, Cdc42EP1–5 [6,7].

Borg proteins were simultaneously discovered by two independent groups as proteins that interact with the Rho GTPases Cdc42 and TC10/RhoQ [6,7]. Using a two-hybrid screen for interactors of TC10, the group of Ian Macara identified three clones containing identical CRIB motifs and flanking regions. Further analysis discovered the ability of these clones to bind active Cdc42 but not to Rac or Rho GTPases. These initial clones were subsequently extended to five putative clones based on sequence homology and their ability to bind Cdc42 in a GTP-dependent manner. They named this new family the Borg (binder of Rho GTPases) proteins (Borg1–5). One of these clones was the previously identified MSE55 [8], which was renamed Borg5. MSE55/Borg5 had already been shown to contain a functional CRIB domain in its N-terminus and to be a non-kinase effector protein of Cdc42 capable of inducing F-actin-based protrusions [9]. Based on these characteristics, the group of Perter Burbelo used the amino acid sequence of MSE55/Borg5 to search for homologous sequences and identified the same five clones [7]. They named the family ‘Cdc42 effector proteins’ or Cdc42EP/CEP, which led to the disparate nomenclature for each member (depending on the classification): MSE55/Cdc42EP1/Borg5, Cdc42EP2/Borg1, Cdc42EP3/Borg2, Cdc42EP4/Borg4 and Cdc42EP5/Borg3. For clarity, here we use the HUGO gene nomenclature to refer to each member (i.e. Cdc42EP1 instead of MSE55/Borg5), but may use the term Borg to refer to the family.

## Structure

**Figure 1.**
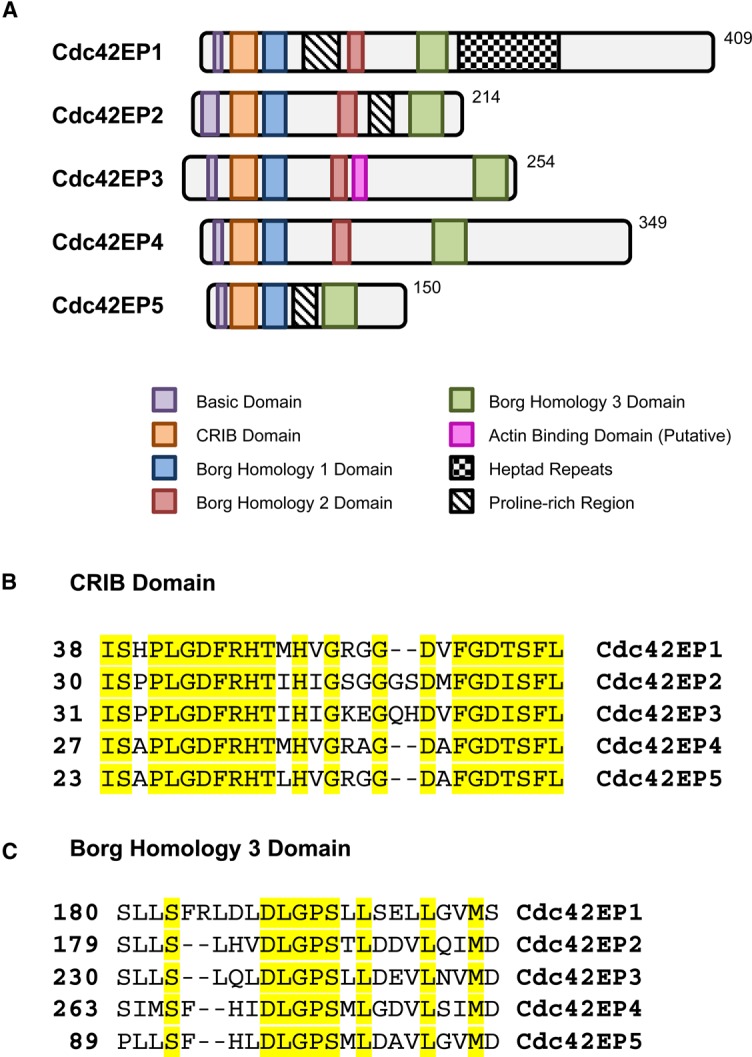
Cdc42EP/Borg family of Cdc42 effector proteins. (**A**) Schematic diagram showing the different domains in all the Borg proteins. (**B**) Direct alignments of the CRIB domains of murine Cdc42EP1–5, with conserved residues highlighted. (**C**) Direct alignments of the BH3 domain of murine Cdc42EP1–5, with conserved residues highlighted.

Borg proteins are relatively small in size, ranging from 150 amino acids (∼15.5 kDa) in Cdc42EP5 to 409 amino acids (∼39 kDa) in Cdc42EP1 (Figure 1A). No family member presents known enzymatically active domains, which suggest that they may exert their biological functions via structural or scaffolding activities. All Borg proteins are characterised by the presence of a highly homologous N-terminal CRIB domain (Figure 1B) [6,7]. In Borgs, the CRIB domain presents an extension at the C-terminus that may mediate its specific binding to active Cdc42 and TC10 but not to Rac1 [6,7], as well as determining part of the particular biological effects of these proteins. Following the CRIB motif, there is a well-conserved short domain that is unique to Borg proteins and that was defined as the Borg Homology (BH) 1 domain [6]. The central and C-terminal parts of the proteins are more divergent, but still present two additional well-conserved regions termed BH2 and BH3. The BH2 domain is absent in the smallest Borg, Cdc42EP5, whereas the BH3 domain is common to all Borgs. The BH3 domain has a central location in Cdc42EP1 and Cdc42EP4, whereas it is localised at the C-terminal parts of Cdc42EP2, Cdc42EP3 and Cdc42EP5 (Figure 1C). The BH3 domain is the only BH domain with a defined molecular role and has been shown to be necessary and sufficient for the specific binding of Borgs to septins [10] (see Box 1). Contrary to the BH3 domain, the roles of the BH1 and BH2 domains are still to be defined. Outside the CRIB and BH domains, sequence divergences emerge. The central regions of Cdc42EP1, Cdc42EP2 and Cdc42EP5 present a proline-rich domain; in addition, Cdc42EP1 contains several heptad repeats that follow the BH3 domain [10]. More recently, it has been described that Cdc42EP3 presents a putative actin-binding region in its central region, C-terminally to the BH2 domain (Figure 1A) [11]. This region presents high homology to the actin bundling region of Anillin. This particular actin-binding region of Cdc42EP3 is not conserved among Borg proteins; however, other Borg members may bind and/or modulate the actin cytoskeleton by alternative mechanisms or via distinct domains.[Table BST-2016-0219TB1]

**Table 1 BST-2016-0219TB1:** The major interactions and functions of Borg proteins

CDC42 effector protein	Interactions	Binding partners	Reported functions	Key references
Cdc42EP1 (Borg5, MSE55)	Cdc42, TC10 (RhoQ), aPKC, ERK2, multiple sites identified by proteomic analysis	SEPT2/6/7	Cell shape regulation, actomyosin contractility, cell migration of endothelial cells and trophectoderm cells, directed and persistent migration, angiogenesis	[6,7,9,10,20,21,33,34]
Cdc42EP2 (Borg1)	Cdc42, TC10, ERK2, ERK3, MK5	SEPT2/6/7	Cell shape regulation and regulator of myogenesis	[6,7,10,20,32,33,37]
Cdc42EP3 (Borg2)	Cdc42, TC10, ERK3, MK5	SEPT2/6/7 F-actin	Cell shape regulation, actomyosin contractility and pathological fibroblast activation	[6,7,10,11,20,31,32]
Cdc42EP4 (Borg4)	Cdc42, TC10, PKCα	SEPT2/6/7, ARHGEF17 (TEM4)	Cell shape regulation, filopodia formation and mammary cell migration	[6,7,10,20,23,24]
Cdc42EP5 (Borg3)	Cdc42, ERK3, MK5	SEPT2/6/7	Cell shape regulation and lamellipodium formation	[6,7,10,20,32]

## Mode of action

At cellular and molecular levels, the function of Borg proteins remains to be clearly defined, but studies thus far indicate relevant roles in cytoskeletal rearrangement, as summarised in Table 1. As with other Cdc42 effector proteins, Borg proteins were initially linked to the regulation of cell shape and early gain-of-function experiments described roles in inducing pseudopodia and F-actin-containing structures in fibroblasts [7,9] and epithelial cells [7]. Shortly after, Joberty et al. [10] elegantly demonstrated that Cdc42EP5 and Cdc42EP2 can bind septins via their BH3 domain and induce septin filament bundling. Further characterisation demonstrated that Cdc42EP5 binds specifically to SEPT6/7 heterodimers or SEPT2/6/7 trimers, but not to septin monomers [20]. This septin-binding property has since been extended to the rest of the members of the family [11,21–23].

**Figure 2.**
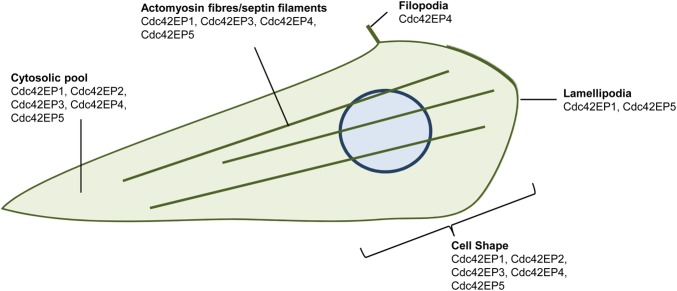
Links between Borg proteins and cytoskeletal regulation and cell shape. Cartoon showing the cellular localisations of Borg proteins. See text for details.

Not surprisingly, most Borg proteins have been shown to form filamentous structures that can co-localise with actin fibres and septin filaments (Figure 2). In normal mammary cells, Cdc42EP4 can co-localise with actin stress fibres and induce filopodia and stress fibre formation [24]. In endothelial cells, Cdc42EP1 aligns with septin filaments and actin stress fibres, and depletion of Cdc42EP1 in this system disrupts both septin and actomyosin fibre assembly [21]. In fibroblasts, Cdc42EP1 and Cdc42EP5 have been reported to induce lamellipodium formation and localise at this structure [4,6,9], hinting towards a possible role in promoting actin polymerisation at the leading edge of migrating cells. On the other hand, in epithelial cells, Borgs generally distribute throughout the cell with no clear specific localisation [6,10].

All these studies suggest a functional interrelationship between Borg proteins, septin networks and the actin cytoskeleton. Some septins (e.g. SEPT9) can bind F-actin and promote the cross-linking of pre-assembled actin filaments [25,26], suggesting that the actin remodelling activity of Borgs might be indirect via septin rearrangements. However, the association between actin and septin networks can also be potentiated by means of adaptor proteins, such as Filamin A [27,28] and Anillin [29], that can enable septin filament assembly following an actin template.

In a recent study investigating the cytoskeletal rearrangements regulating the emergence of pathologically activated fibroblasts (i.e. cancer-associated fibroblasts or CAFs), we documented the ability of Cdc42EP3 to bind both F-actin and septins, and to act as an adaptor protein that reinforces both networks [11]. Using super-resolution microscopy, we observed that Cdc42EP3 forms an intricate filamentous network in CAFs that co-localised with septin filaments. These Cdc42EP3–septin filaments clearly aligned but not exactly overlapped with F-actin and also formed connections between actin fibres. Our study demonstrated that Cdc42EP3 depletion reduced the filamentous septin network in CAFs [11], an effect that was also detected after blocking stress fibre formation by depleting the well-known actin remodelling proteins DIAPH1&3 [30]. Conversely, disrupting the septin network amply affected the formation of actin stress fibres. These cytoskeletal defects extensively altered the actomyosin contractility machinery, leading to a diminished ability of CAFs to generate and sense forces. The main consequence was a drastic inactivation of mechano-transduction signalling (i.e. FAK, Src) and transcription (i.e. YAP), all processes dependent on intact actomyosin fibres. In CAFs, Cdc42EP3 appears to allow the template function of actin needed for septin polymerisation, as well as to enhance the F-actin cross-linking activity of septins.

Interestingly, Cdc42EP1 presents a similar pattern of localisation in endothelial cells where Cdc42EP1 depletion also leads to loss of cellular contractility and both persistent and directional migration [21], suggesting that these particular functions may be shared by other Borg proteins. Other modes of action may be more divergent. These most probably depend on primary structure differences as well as particular patterns of tissue expression that dictate specific functions and binding partners. For example, Cdc42EP4 is exclusively expressed in Bergmann glia in the cerebellum where it localises beneath specific membrane domains. In this system, Cdc42EP4 forms complexes with septin hetero-oligomers, which interact with and modulate a subset of glutamate transporters [23]. On the other hand, in mammary epithelial cells under protein kinase Cα (PKCα) stimulation, Cdc42EP4 dissociates from Cdc42 and binds the Rac-GEF (guanine nucleotide exchange factor) ARHGEF17/TEM4, leading to Rac1 activation and increased motility [24].

## Regulation

As other CRIB-containing Cdc42 effector proteins, Borg proteins are mainly regulated by a specific interaction with active Cdc42-GTP [6,7]. Except for Cdc42EP5, Borg proteins can also bind activated TC10/RhoQ [6,7], but this regulation is largely understudied and will not be reviewed here. Seminal studies demonstrated that the cellular responses elicited by Borg proteins are Cdc42-dependent and do not require Rac1 [6,9]. Indeed, Cdc42-binding defective mutants present diminished activities and no longer induce the characteristic morphological and cytoskeletal rearrangements of their wild-type counterparts [7,9,31]. Recently, we reported that a Cdc42-binding defective mutant of Cdc42EP3 (Cdc42EP3-IS/AA) has lower binding affinity towards actin and septins in co-immunoprecipitation assays [11]. This mutant cannot form the characteristic filamentous network in CAFs and shows a disperse localisation in the cytosol [31]. In addition, Cdc42EP3-IS/AA appears to act as a dominant-negative as both actin stress fibres and particularly septin filaments are amply diminished in CAFs following Cdc42EP3-IS/AA expression. Similar results were observed in wild-type Cdc42EP3 after ectopic expression of the dominant-negative mutant Cdc42-N17 [31]. However, ectopic expression of the constitutively active mutant of Cdc42-V12 does not potentiate Cdc42EP3 functions. On the contrary, constitutive activation of Cdc42 leads to a massive redistribution of Cdc42EP3 to Cdc42-V12-enriched vesicles. As a result, the characteristic filamentous pattern of Cdc42EP3 and septins is lost and actin stress fibres are reduced [31]. Similar results were previously reported for Cdc42EP5, where constitutive activation of Cdc42 inhibits Cdc42EP5 binding to septins and a resultant loss of septin filaments [10]. The available data support a model whereby interaction with active Cdc42 enables the correct positioning of Borg proteins in defined subcellular localisations where they can associate with specific binding partners (i.e. septin and/or actin) and perform their function. In this model, the adequate cycling of Cdc42 between its GDP-bound and GTP-bound forms is required for: (i) the ideal positioning of the Borg–Cdc42 complex in specific regions of the cell and/or (ii) releasing Borg proteins from the complex to allow further functional interactions.

The activity of Borg proteins may also be regulated by alternative mechanisms (Table 1). Cdc42EP2, Cdc42EP3 and Cdc42EP5 are direct substrates of the atypical MAP kinases ERK3 and MK5, and have been suggested to be relevant in the regulation of neuronal cytoskeleton and dendritic spine formation [32]. *In vitro* chemical–genetic screens have also identified Cdc42EP1 and Cdc42EP2 as ERK2 substrates [33]. Cdc42EP1 has been shown to interact with atypical PKC in mouse differentiating trophectoderm cells leading to increase cell motility [34]. In addition, Cdc42EP4 is a substrate of PKCα in human breast MCF-10A cells. PKC-mediated phosphorylation reduces Cdc42EP4 affinity to Cdc42 allowing binding to ARHGEF17 and leading to Rac1 activation and cell migration [24]. These findings hint at a more complex scenario as they indicate that Borg proteins may functionally interact with other Rho GTPases. Indeed, seminal studies on Borgs already suggested a possible role of Borg proteins in partially inhibiting RhoA function and demonstrated that Borg phenotypes can be diminished by activation of RhoA signalling [6]. Nevertheless, further studies are required to fully understand these interactions and to delineate the exact regulatory mechanism/s controlling Borg function.

## Borgs in development and disease

As opposed to other Cdc42 effectors, Borg genes are only found in vertebrates and no orthologues have been found in bacteria, yeast, worms or flies, suggesting a late emergence during evolution. In part, this may account for the current lack of functional and biological information on these proteins. Nevertheless, functional homologous of Borg proteins are starting to emerge. For example, the yeast Cdc42 effectors Gic1 and Gic2 have shown to possess similar functions such as septin filament assembly and cell polarity [35,36].

Thus far, it appears that Borg proteins share similar primary structures, regulatory mechanisms and modes of action, suggesting that part of their biological relevance may be determined by their distinct patterns of expression. Little is known about how Borg gene expression varies throughout development and adult life, but it is evident that they are differentially expressed in tissues. *Cdc42EP1* expression was initially found to be restricted to endothelial and bone marrow stromal cells [8], with high expression levels in the microvasculature of the heart and brain [21]. *Cdc42EP2* and *Cdc42EP3* are preferentially expressed in the heart with reduced levels in lung, kidney and skeletal muscle, whereas *Cdc42EP5* was specifically detected in skeletal muscle [6]. As opposed to *Cdc42EP3* and *Cdc42EP5*, *Cdc42EP2* was also detected in brain tissue [6], where expression of *Cdc42EP4* has also been recently reported, particularly in the cerebellum [23].

Some crucial functions for Borgs in development have emerged from Borg knockout mice, with some roles also interestingly coinciding with their expression patterns. Using a Cdc42EP1-null mouse, Zheng and colleagues identified a critical function for Cdc42EP1 in heart development and angiogenesis. Cdc42EP1-knockout mice showed reduced viability, as well as reduced capillary density and thinner ventricular walls [21]. Functional characterisation of cardiac endothelial cells from these mice indicated a key role of the Cdc42EP1–septin axis in promoting angiogenesis by regulating persistent directional migration through spatial control of actomyosin contractility. Another study by the same group also demonstrated that Cdc42EP1 also plays a role in regulating the migration and sorting of trophectoderm cells during embryogenesis [34]. In sharp contrast, ectopic expression of Cdc42EP2 has been reported to lead to a decrease in differentiation of mouse myoblasts, whereas Cdc42EP2 knockdown increases myogenesis [37]. In the cerebellum, Cdc42EP4 forms complexes with septins, which in turn interact with the glutamate transporter GLAST [23]. In Cdc42EP4 knockout mice, GLAST dissociates from septins resulting in aberrant glutamate buffering and clearance. These defects are accompanied by mild neural dysfunctions and impaired motor co-ordination/learning.

Even more obscure are the links between Borg proteins and disease. We recently demonstrated a key role of Cdc42EP3 in the emergence of CAFs, stromal cells that promote tumour progression and therapeutic resistance [38,39]. In breast cancer, Cdc42EP3 is up-regulated early in fibroblast activation and co-ordinates actin and septin rearrangements. This results in a potentiated response to chemical and mechanical stimulation that leads to a tumour-promoting CAF phenotype [11]. More importantly, Cdc42EP3 is required for most of the hallmarks of CAFs, notably extracellular matrix remodelling and promotion of angiogenesis, cancer cell growth and invasion. Whether Borgs play any other roles in tumorigenesis is still to be determined. Interestingly, the Burbelo group observed that Cdc42EP5 overexpressing keratinocytes present increased stress fibres and reduced E-cadherin expression at adherens junctions, indicative of an epithelial-to-mesenchymal transition or EMT, a hallmark of cancer metastasis [7]. In addition, Cdc42EP3 expression is also up-regulated during a TGF-β-induced EMT in human keratinocytes [40], and numerous studies are reporting the differential expression of Borgs in tumoural settings. Further investigation on these interesting observations is definitely required.

## Conclusion

Clearly, there is still much to uncover about the biological functions of Borg proteins. In that respect, the lack of *in vivo* models has hindered progress. Nevertheless, recent findings have underlined that their relevant role in angiogenesis and pathological activation of tumour stroma, which coupled with an improved understanding of their mode of action and regulatory mechanisms, offers a promising avenue for new research. In particular, the identification of Borg proteins as key regulators of septin organisation in mammalian cells may assist in the understanding of septin functions, which is attracting a lot of attention nowadays.

## Abbreviations

BH: Borg homology
Borg: binder of Rho GTPases
CAF: cancer-associated fibroblast
CDC42EP/CEP: Cdc42 effector protein
CRIB: Cdc42/Rac interactive binding
DIAPH: diaphanous related formin
EMT: epithelial-to-mesenchymal transition
FAK: focal adhesion kinase
GEF: guanine nucleotide exchange factor
HUGO: Human Genome Organisation
MAP: mitogen-activated protein
PKCα: protein kinase Cα
TGF-β: transforming growth factor beta
YAP: yes-associated protein
